# Health and social care leaders’ and employees’ perceptions of remote leadership and the associated factors

**DOI:** 10.1108/LHS-01-2024-0010

**Published:** 2024-07-05

**Authors:** Anja Terkamo-Moisio, Elsa Paronen, Arja Häggman-Laitila, Johanna Lammintakanen

**Affiliations:** Department of Public Health, University of Helsinki, Helsinki, Finland and Department of Nursing Science, Faculty of Health Sciences, University of Eastern Finland, Kuopio, Finland; Department of Health and Social Management, Faculty of Social Sciences and Business Studies, University of Eastern Finland, Kuopio, Finland; Department of Nursing Science, Faculty of Health Sciences, University of Eastern Finland, Kuopio, Finland and City of Helsinki Social and Health Services, Helsinki, Finland; Department of Health and Social Management, Faculty of Social Sciences and Business Studies, University of Eastern Finland, Kuopio, Finland

**Keywords:** Digitalization, Remote leadership, Health and social care leaders, Health and social care personnel, Survey

## Abstract

**Purpose:**

The purpose of this study was to describe health and social care leaders’ and employees’ perceptions of remote leadership and the associated factors.

**Design/methodology/approach:**

A total of 45 leaders and 177 employees from one Finnish health and social care organization completed an electronic questionnaire between October and November 2020. The questionnaire included questions related to background information, along with structured and open-ended questions addressing remote leadership and the associated factors. The collected quantitative data was analyzed with statistical methods, while inductive content analysis was used to analyze the qualitative data.

**Findings:**

Remote leadership emerged as a developing form of leadership that was part of everyday life at a regional health and social care organization. However, it was also considered by some as a distanced and authoritarian form of leadership that reduced communication to a one-way flow of information. Remote leadership and digitalization in health and social care were generally perceived positively, especially among higher educated participants and those working mainly in a remote context. However, digitalization was also perceived as a burden and remote leadership as a source of uncertainty at work, especially among lower educated participants and those who worked mainly in traditional contexts.

**Originality/value:**

This study expands the little-researched area and provides insights that can be used to further develop remote leadership and the related education.

## Introduction

Remote leadership has become more common in the health and social care sector, especially during and after the COVID-19 pandemic (Ameel *et al.*, 2022; Kiljunen *et al.*, 2022; Hurmekoski *et al.*, 2023). Aside from the move to remote work that occurred during the COVID-19 pandemic, the prevalence of remote leadership has been brought about by increased globalization and advances in various digital technologies. Moreover, structural reforms of the health and social care sector have resulted in an increased number of decentralized organizations (Terkamo-Moisio *et al.*, 2022). Remote leadership can be defined in various ways, for instance, via terms such as digital leadership, e-leadership, or e-HRM (Torre and Sarti, 2020; Klus and Müller, 2021; Terkamo-Moisio *et al.*, 2022). A geographical and/or temporal distance between the leader and employees, along with the utilization of information technology solutions for interactions at work, are aspects that are most commonly shared between different definitions (Cortellazzo *et al.*, 2019; Tigre *et al.*, 2023). In the current study, remote leadership is defined as a situation in which a health and social care leader leads a geographically dispersed team with the support of information technology in a way that is aligned with socioemotional aspects and organizational guidelines (Cowan, 2014; Sharpp *et al.*, 2019). In line with previous literature (Avolio *et al.*, 2014; Terkamo-Moisio *et al.*, 2022; Tigre *et al.*, 2023), remote leadership is seen here as a social process that is technology-mediated and changes individuals’ behaviors, engagement and attitudes.

The digitalization of health and social care covers the advent of remote leadership as well as the implementation of various products and solutions that improve processes and increase the effectiveness of care services (Vandresen *et al.*, 2022). This includes mobile and remote health technology, cloud-based services, telemonitoring tools or health records, electronic appraisal tools for human resource management and solutions for safety and quality management, among others (Sharpp *et al.*, 2019; Martins *et al.*, 2020). Even though previous literature has widely highlighted the positive aspects of digitalization in health and social care (Sharpp *et al.*, 2019; Martins *et al.*, 2020; Ameel *et al.*, 2022; Vandresen *et al.*, 2022), leaders have nevertheless reported feeling overwhelmed as a result of the various digital tools they now use in their work (Sharpp *et al.*, 2019). Furthermore, successful adaptation to new technologies has been found to be associated with the age and digital skills of a health and social care leader or employee (Vandresen *et al.*, 2022). In other words, it is assumed that younger generations are more attuned to digital solutions than older generations (Chicca and Shellenbarger, 2018). As such, it is unsurprising that a health and social care leader’s digital skills are crucial for their ability to lead a remote team (Ameel *et al.*, 2022; Kiljunen *et al.*, 2022; Terkamo-Moisio *et al.*, 2022; Hurmekoski *et al.*, 2023). Previous literature describes the various information technology competences that affect the success of remote leadership, including those requiring further training and education (Sharpp *et al.*, 2019; Ameel *et al.*, 2022; Kiljunen *et al.*, 2022; Hurmekoski *et al.*, 2023). This also highlights how leaders serve as change agents in an organization’s digital culture (Cortellazzo *et al.*, 2019).

Scientific knowledge outside of health and social care highlights the leaders’ personal traits and competences, such as availability, empathy and willingness to enhance employees’ autonomy, as prerequisites of successful remote leadership (Terkamo-Moisio *et al.*, 2022). These characteristics are prominent in the relational leadership styles, such as transformational, authentic or ethical leadership, that have been found to be associated with several positive staff and patient outcomes within health and social care (Hult *et al.*, 2023). To our current knowledge, this topic has not been researched in the context of remote leadership in health and social care. However, knowledge of remote leadership in other areas has demonstrated that a leader’s situational-based ability to use different leadership styles has positive effects on success in the remote context (Terkamo-Moisio *et al.*, 2022).

Aside from leadership styles, reciprocal trust between the leader and employees is another key aspect of remote leadership that – according to prior research – may decrease in the remote context (Kiljunen *et al.*, 2022; Terkamo-Moisio *et al.*, 2022; Tigre *et al.*, 2023). According to the previous review by Terkamo-Moisio *et al.* (2022), trust-building in a remote context is difficult due to the lack of natural human interactions and reactions. Thus, remote leaders should proactively invest time to build and maintain trust with their employees. Health and social care leaders who operate in a remote context can demonstrate trust by avoiding unnecessary control over employees and being accessible despite the distance (Hurmekoski *et al.*, 2023). Trust has been found to enhance team performance. Therefore, it is central to achieving organizational goals (Terkamo-Moisio *et al.*, 2022; Tigre *et al.*, 2023). Furthermore, it has been stated that employees who work in a remote context need the trust of their leaders to independently carry out their work (Hurmekoski *et al.*, 2023).

Remote leaders are expected to initiate regular formal and informal communication with employees, as well as create effective communication channels (Kiljunen *et al.*, 2022; Terkamo-Moisio *et al.*, 2022). This is particularly significant in the context of health and social care, which is a human-intensive sector that involves interactions and face-to-face contact on a daily level (Ameel *et al.*, 2022; Kiljunen *et al.*, 2022; Terkamo-Moisio *et al.*, 2022). Numerous studies have highlighted how face-to-face contact is still needed in the remote setting, for example, during conflict situations or for the onboarding of new employees (Ameel *et al.*, 2022; Kiljunen *et al.*, 2022; Terkamo-Moisio *et al.*, 2022; Hurmekoski *et al.*, 2023). Successful communication has been found to positively impact team culture and the leader–employee relationship. Furthermore, the quality of communication has been shown to be related to the level of reciprocal trust. Therefore, remote leaders should strive to create a psychologically safe environment where difficult themes can be addressed via open and honest communication. It is important to note that physical distance does not always influence the effectiveness of communication; therefore, the focus should always be on the quality of communication (Terkamo-Moisio *et al.*, 2022).

Despite growing research interest in remote leadership in the context of health and social care in the past few years, there is a general paucity of knowledge in this area (Kiljunen *et al.*, 2022; Terkamo-Moisio *et al.*, 2022; Tigre *et al.*, 2023). This study complements the existing knowledge base by examining health and social care leaders’ and employees’ experiences and perceptions of remote leadership and its associated factors.

## Methods

This study used a cross-sectional survey design that involved both qualitative and quantitative questions and was reported according to the STROBE checklist (Von Elm *et al.*, 2007).

### Aim and research questions

The aim of the study was to describe health and social care leaders’ and employees’ perceptions of remote leadership, along with the associated factors, by addressing these research questions:

1. What kinds of perceptions do health and social care leaders and employees have of remote leadership?

2. Which factors are associated with health and social care leaders’ and employees’ perceptions of remote leadership?

### Participants and recruitment

To reduce sampling error, a purposive, total sampling technique was used in this research (Burns and Grove, 2009). The target group (*N* = 1,405) consisted of employees (*N* = 1,329) and leaders (*N* = 76) from one health and social service organization in Finland. In addition to employment in the organization, participants needed to have appropriate skills in the Finnish language, which was used in the electronic questionnaire. The organization has integrated service provision to serve a quite large geographical area (*ca* 40,000 residents), and it provides health and nursing services and social welfare services, along with environmental health care, veterinarian welfare and environmental protection and health surveillance services to citizens. After receiving an organizational research permit, an invitation to participate in the research was sent to the organization’s contact person, who forwarded it on to all employees of the organization. The study invitation included written study information and a link to the questionnaire. Furthermore, to ensure that participants had the possibility to ask for further information about the research, the researchers’ contact information was included in the study information. The organization’s contact person sent two email reminders to the employees during the data collection period.

### Data collection

The data was collected through an electronic survey conducted between October and November 2020. The questionnaire was designed for this study as a part of the “More Remotely – work in health and social care is changing” project within an expert group (*N* = 6) encompassing the areas of nursing science, health management sciences, design thinking, work-related wellbeing and work supervision. Existing literature (e.g. Hahm, 2017; Sharpp *et al.*, 2019*)* and previously nationally validated instrument were used as the foundation of the questionnaire that was pretested for feasibility by health and social care professionals (*N* = 61) prior to the data collection. Pretesting did not result in any changes being made to the questionnaire.

The questionnaire included structured and open-ended questions regarding remote leadership, remote working culture, as well as respondents’ background information. Participants were asked about their gender, age and highest level of education as background information. In addition, they were asked for information regarding their current professional status (e.g. if they worked in a management position) and remote work (e.g. were they working mainly in a remote context).

The participants were asked to evaluate their level of agreement with statements (*N* = 7) related to remote leadership (e.g. *“*Physical distance has not weakened the trust between my manager and me”) and remote working culture (e.g. “Remote leadership does not weaken the working atmosphere”) with a five-point Likert scale (1 = completely agree – 5 = completely disagree). Participants who worked in management positions were asked to express their level of agreement with additional statements (*N* = 12) regarding remote leadership (e.g. “I treat my employees equally regardless of physical distance” and “Remote leadership makes me feel secure as leader”) and remote working culture (e.g. “Remote leadership has not increased insecurity among employees”) with a five-point Likert scale (1 = completely agree – 5 = completely disagree). In addition, the questionnaire contained an open-ended question: “What are your perceptions of remote leadership, and how should it be developed in your opinion?” This enabled participants to freely express their perceptions about remote leadership.

### Data analysis

The quantitative data were analyzed by using IBM SPSS Statistics 29 (SPSS Inc., Chicago, IL) for Windows. Descriptive statistics (frequencies and percentages) were used to describe participants’ demographic information. Participant age was recoded into three groups (≤40 years, 41–55 years and ≥50 years). Similarly, educational level was recoded into three groups (master’s degree or higher, bachelor’s degree or equivalent and secondary education or other). Furthermore, participants’ answers to the question if they worked mainly in a remote context were recoded into two groups (remote leader and remote employee = yes, does not apply to me = no). Also, the participating leaders were divided into two groups based on the number of remote employees they lead (no remote employees = no, 2–50 remote employees = yes). Finally, the answers provided on the Likert scale were recoded into three groups, namely, agree (including the responses “completely agree” and “mostly agree”), neutral (including the answer “neither agree nor disagree”) and disagree (including the responses “completely disagree” and “mostly disagree”).

Descriptive statistics were used to present an overview of the answers given to the statements. It should be noted that among the group of leaders, fewer than 10 respondents provided responses to some of the questions; hence, the results are only presented as percentages. Thereafter, Pearson’s correlation was used to explore associations between the statement and participants’ demographic information. Correlation was seen as weak by r-values ≤0.3, moderate by r-values >0.3–≤0.5 and strong by r-values >0.5 (Field, 2013). The non-normal distribution of the data was confirmed by the Kolmogorov–Smirnov test, thus Kruskal–Wallis and Mann–Whitney U-tests were used for examination of differences between groups. In evaluation of statistical significance, p-values <0.05 were considered as significant and p-values <0.001 very significant (Field, 2013).

The answers to open-ended questions were analyzed by inductive content analysis (Elo and Kyngäs, 2008). In the first phase, the collected responses were read through multiple times to gain an overview of the content. Thereafter, original expressions (*N* = 77) were condensed and grouped by content into subcategories that were inductively named (for example, “One-way flow of information”). In the next phase of abstraction, the subcategories were grouped into upper categories based on similar content and named (for example, “Experiences of remote leadership”). During the final phase of analysis, one main category was named as “Participants’ perceptions of remote leadership and its development” based on the content of the upper categories (Elo and Kyngäs, 2008; Elo *et al.*, 2014). This analysis was conducted by two members of the research team (A.T.-M. and J.L.), who independently analyzed the qualitative data. The results were compared and discussed until a consensus was reached.

### Ethical considerations

The research followed the guidelines of the Finnish National Board on Research Integrity (TENK) and was in line with Finnish legislation; as such, research ethical approval was not required (Finnish National Board on Research Integrity TENK, 2020). A research permit was obtained from the target organization due to organizational customs. Participants were informed in writing that participation was voluntary, and that they had the right to withdraw from the study at any time. In addition, this information outlined the way in which participants would take part in the survey, as well as the possible risks and benefits of participation. All participants gave their informed consent on the first page of the questionnaire (Finnish National Board on Research Integrity TENK, 2020).

## Results

### Description of participants

A total of 45 leaders (response rate: 59%) and 177 employees (response rate: 13%) completed the questionnaire (Table 1). Participants were mainly female (93%), and over half (51%) held a bachelor’s or equivalent degree. The mean age of the participants was 47 years (range 21–67, SD = 11.4). Over a third (39%) of the participants worked mainly in a remote context, either as remote leader (7%) or remote employee (32%). The total number of leaders’ employees ranged from zero to 350 (mean = 28, SD = 59.10), and the number of their employees working mainly in a remote context varied between zero and 50 (mean = 6, SD = 10.10).

### Participants’ perceptions of remote leadership

The analysis of qualitative data revealed three upper categories, namely, *appearance of remote leadership, experiences of remote leadership* and *development of remote leadership* (Table 2).

#### Appearance of remote leadership.

Some of the participants described remote leadership as *successful and functioning leadership*, which included references to the leader being accessible and employees not feeling forgotten. Participants mentioned that traditional leadership had shifted into an electronic form and that leadership practices were functioning well. Other participants, however, described the remote leadership they experienced as *distanced and authoritarian leadership.* In these latter descriptions, the employees were sometimes unsure who their leader was or if their leader was accessible. These respondents also pointed out that the leaders did not know their employees or were not invested in remote leadership. Moreover, some respondents shared that they felt the leadership to be inherently unjust, including stringent top-down guidelines as well as unrealistic instructions from their managers. The participants elaborated that this kind of remote leadership appears cold and authoritarian. Respondents also shared how remote leadership was sometimes *reduced to virtual meetings.* In addition, participants felt that the leader needs to know what type of work is carried out by the unit and employees which they are responsible for. Furthermore, the respondents pointed out that remote leadership requires strong self-regulation, with some feeling that remote leadership is *inappropriate for their own unit*. In contrast, other participants regarded remote leadership as a *developing form of leadership* that is currently being refined:

Remote leadership functions when the leader invests in it as if he/she would be physically present. At the moment, my leader has no knowledge about the clinical work in the unit and he/she is not able to think like an employee or see how stressful the work actually is.

I do not actually know who my leader is anymore.

Leadership was rarely present in remote work, and only visible in meetings once a week.

[Remote leadership] is searching for its forms. This requires open, multifaceted discussion about how it works in practice.

#### Experiences of remote leadership.

The participants shared that they felt as though the work they do had not changed following the increasing implementation of remote leadership; as such, remote leadership was considered as a *part of daily work life*, especially in decentralized organizations and following the COVID-19 pandemic. However, some participants mentioned the *one-way flow of information* and described situations in which information or instructions were given to employees without the possibility of giving feedback. Furthermore, participants experienced remote leadership as *electronic information that is challenging to access.* They felt that leaders should ensure that the information reaches their employees, as it may not necessarily be readily available, for example, on the organization’s intranet:

Remote leadership is part of everyday life in a regional organization.

Issues are announced in e-mails and they are not processed together.

Leaders should ensure the transfer of information, as all of the information is not necessarily on the organization’s intranet.

#### Development of remote leadership.

The collected descriptions revealed participants’ expectations of *active and leader-driven remote leadership*. Participants pointed out that remote leaders should show an interest in their employees, for example, by asking them how they are doing instead of just saying that they may contact the leader when necessary. It is thus unsurprising that the participants highlighted *the significance of face-to-face meetings.* The participants felt that these types of physical meetings should be regularly organized, especially if there are new members in the team. Furthermore, participants pointed out that building trust requires personal meetings and stated that remote leadership may not replace live meetings. In addition, participants identified *regular interaction and information* as an area that requires development. They expressed the hope that monthly meetings would be reinstated and felt that certain issues remain unresolved in many units because the leader is not physically present to address them. Participants also pointed out that all leaders do not have the abilities required for effective remote leadership. Moreover, the descriptions highlighted how digital skills vary among employees, with suggestions that the *education of leaders and employees* can develop remote leadership:

There does not always need to be actual work-related issues, the leader can just ask “how are you” and “how are you holding up?”

Meeting face-to-face is enormously important for building a trustful relationship.

Mostly I miss the regularity in contact and communication.

Remote leadership can be developed by educating the leaders, as all of them do not have the abilities required for this task.

### Health and social care employees’ and leaders’ perceptions of remote leadership with associated factors

The majority (67.9%) of participants considered the importance of management to have increased with the increased prevalence of remote leadership (Table 3), which was not statistically significantly associated with participants’ demographic information. Nearly half (43.9%) felt that the spread of digitalization burdens them as an employee, which is statistically significantly associated with participants’ educational level (*r* = −0.161, *p* = 0.009) and managerial position (*r* = −0.157, *p* = 0.023). The comparison of groups revealed that participants with a master’s degree or higher to be less burdened than those in other educational groups (*p* = 0.005). Those participants who worked in managerial positions (*p* = 0.024) were also less burdened. Almost half (49%) of the participants were neutral toward remote leadership’s positive effect on the working atmosphere. A positive correlation was found to the highest education of the participant (*r* = 0.140, *p* = 0.049), which was confirmed by comparison of the groups showing participants with a master’s degree or higher reporting statistically significantly more positive perception than those with a bachelor’s degree or equivalent (*p * = <0.001).

A minority (21%) of the participants reported remote leadership to have weakened the trust between their manager and themselves, whereas nearly a third (32%) were neutral toward this statement. Positive correlations were found between this statement and participants’ educational level (*r* =−0.188, *p* = 0.008), managerial position (*r* = −0.200, *p* = 0.004), and remote context (*r* = −0.309, *p* = <0.001). Comparison of groups revealed that those participants with secondary education or other agreed with the statement about trust more often than those with a master’s degree or higher (*p* = 0.016). Furthermore, participants in a managerial position (*p* = 0.004) and those who worked mainly in remote contexts (*p*=<0.001) disagreed more often with this statement about trust than employees or those working less frequently in a remote context.

Over half (51%) perceived the spread of digital communication tools not to weaken the interactions with their manager, a perception that was associated with participants’ educational level (*r* = 0.155, *p* = 0.026) and managerial position (*r* = 0.198, *p* = 0.004). Participants with a master’s degree or higher disagreed more with the statement than participants in other educational groups (*p* = 0.010). Similarly, those who worked mainly in remote contexts had a more negative perception of the statement than others (*p* = 0.031). In line with this, over half (54%) perceived that remote leadership did not weaken their opportunities to be in contact with their manager. A majority (66%) of the participants did not feel uncertain about the continuation of their employment as remote leadership increases. This was associated with participants’ educational level (*r* = −0.313, *p* = <0.001) and the context in which they mainly work (*r* = −0.243, *p* = 0.001). Comparison of groups showed participants with a master’s degree or higher to be least uncertain (*p* = <0.001). Furthermore, those who worked mainly in remote contexts disagreed more with the statement regarding work uncertainty than others (*p* = <0.001).

### Health and social care leaders’ perceptions of remote leadership with associated factors

The majority (87.2%) of leaders who participated reported mutual trust between themselves and their employees (Table 4). Statistically significant correlations were found in this regard with leaders’ education (*r* = −0.385, *p* = 0.015) and the number of their remote employees (*r* = 0.485, *p* = 0.019). Leaders with a master’s degree or higher reported more neutral opinions than leaders in other educational groups (*p* = 0.038). Most (61.5%) of the leaders did not report physical distance to weaken the bonds of trust between them and their employees, which showed a positive correlation (*r* = −0.403, *p* = 0.025) with the number of employees. A fifth of the leaders (20.5%) did not agree with the statement indicating common rules of the game for remote leadership in their organization, and an additional 20.5% were neutral in this regard (Table 4). A majority (73.7%) of the leaders found their leadership skills to meet the requirements of remote leadership, and most of them (76.9%) did not feel insecure as leader due to remote leadership. However, over a fourth (26.3%) of the leaders agreed, and nearly a third (31.6%) were neutral regarding the statement indicating increasing insecurity among employees due remote leadership. Leaders’ opinions of employees’ insecurity showed a positive correlation (*r* = −0.484, *p* = 0.002) with their age. Leaders in the age group ≥56 years disagreed with the statement more than the leaders in the age groups 41–55 years or ≤40 years (*p* = 0.027). Over half (64.1%) of the leaders reported that increasing digitalization made their work as leader easier. This correlated positively with leaders’ education (*r* = 0.363, *p* = 0.023). Nearly a third (30.8%) of leaders felt burdened by increased digitalization, an opinion that had a positive association with leaders’ education (*r* = −0.482, *p* = 0.002). Leaders with a master’s degree or higher felt more burdened by increased digitalization than those with a bachelor’s degree or equivalent (*p* = 0.008). A majority (74.4%) of the leaders felt that they are sufficiently available to their employees, whereas nearly a fifth (17.9%) agreed and a third (33.3%) were neutral about the statement indicating remote leadership communication tools to weaken interactions. Positive correlation (*r* = −0.409, *p* = 0.010) was found between this statement and leaders’ education. In addition, leaders with a master’s degree or higher disagreed most with the statement indicating that remote leadership communication tools weaken interactions, followed by a bachelor’s degree or equivalent and secondary education or other (*p* = 0.043).

## Discussion

This study described health and social care leaders’ and employees’ perceptions of a little-researched area – remote leadership and the associated factors. The timepoint of the data collection in the second half of the first year of the COVID-19 pandemic reveals the leaders’ and employees’ perspectives at a time when remote leadership and working were becoming increasingly common, and not just in the health and social sector (Kiljunen *et al.*, 2022). Thus, our results revealed insights that could be beneficial to various organizations in a range of fields, future research, and the education of health and social care professionals.

Despite the participants’ positive perceptions of remote leadership, some of them perceived it as a distanced and authoritarian form of leadership that lacked innate justice and possibilities for participation. This was further emphasized by some participants’ perceptions of weakened levels of trust and interactions. However, at the same time, the increased prevalence of remote leadership was perceived to increase the importance of management. Understanding this contradiction is essential from the perspective of leadership and management research and raises the question of if the rather multifaceted and people-centered leadership in health and social care is in fact narrowing down to task-oriented management discharged in a remote context. Furthermore, the described experiences in this regard contradict previous results about how leadership must consider socioemotional aspects (Cowan, 2014; Sharpp *et al.*, 2019). This warrants attention in relevant organizations and in future research. Organizations should provide health and social care leaders with ample opportunities to improve their leadership skills, as it cannot be assumed that the skills associated with traditional face-to-face leadership are directly transferrable to the remote context (Cortellazzo *et al.*, 2019). In addition, organizations should build and enhance psychologically safe environments in which negative perspectives may be brought out for discussion (Terkamo-Moisio *et al.*, 2022). Furthermore, organizations should develop means and structures that enhance employees’ participation in the remote context to strengthen the human orientation and avoid the narrowing of leadership. The finding that the participating leaders and employees mainly considered remote leadership as a developing form of leadership highlights the significance of its conceptual examination in future research.

In accordance with the previous literature (Ameel *et al.*, 2022; Kiljunen *et al.*, 2022; Terkamo-Moisio *et al.*, 2022; Hurmekoski *et al.*, 2023), the data collected here highlighted the need for face-to-face meetings. This finding may be linked to the human-intensive nature of health and social care (Terkamo-Moisio *et al.*, 2022) and the fact that remote leadership is a rather new development in this setting. The previously suggested improvement of participation in a remote context may be considered as one means to improve the employees’ sense of community and belonging. This finding may also support the suggestions of previous literature, in that hybrid leadership will become a clear trend in health and social care (Ameel *et al.*, 2022; Hurmekoski *et al.*, 2023).

In the current study, over half of the leaders agreed that guidelines for remote leadership existed in their organization. This in itself can be considered a positive result, as previous literature has highlighted the lack of joint guidelines (Ameel *et al.*, 2022; Terkamo-Moisio *et al.*, 2022; Hurmekoski *et al.*, 2023). Nevertheless, this result was not as evident to nearly half of the participating leaders, which highlights the issue of how accessible information actually is within organizations, along with the possible asymmetry of existing information. It is important to note that the burden of the COVID-19 pandemic was particularly evident among health and social care leaders at the time of data collection. However, not all of the leaders worked mainly in a remote capacity, which, despite the lack of statistical significance in the differences between their perceptions, may indicate the lack of relevance of these guidelines for some of the participants. These may have influenced the results presented in this article, and the subject thus warrants further research.

Interestingly, nearly half of the participants reported that they perceived the spread of digitalization as a burden, even though more than half of the leaders stated that it has made their work as a leader easier. Previous research has found increasing digitalization to be overwhelming (Sharpp *et al.*, 2019), which may partly explain this result. A further possible explanation for the reported result may lie in the diversity of digital skills reported in the literature (Ameel *et al.*, 2022; Kiljunen *et al.*, 2022; Hurmekoski *et al.*, 2023). The latter may be supported with the result indicating higher educated participants to be less burdened, as with the spread of digitalization, digital tools have become a central part of, for example, university-level education. On the other hand, in future research, attention should be paid to possible age differences, as younger generations, especially so-called Generation Z, have been described as confident with digital solutions because they use them so widely in everyday life (Chicca and Shellenbarger, 2018). For instance, it could be possible that presumptions about younger leaders and professionals having stronger digital skills could lead to greater expectations for support from colleagues and thus increase the burdens.

Our analysis of the collected data identified contradictory perceptions of work-related uncertainty among the participants. In general, a minority of the participants reported uncertainty about their employment as remote leadership increases. More highly educated participants and those working mainly in remote contexts reported less work-related uncertainty, highlighting the positive effect of education and experience gained in the remote context. However, a fourth of the leaders perceived their employees to have work-related uncertainty because of increasing remote leadership. Interestingly, this perception was more common among the younger leaders. It is possible that the increasing experience connected with age or working as a leader may influence this perception. However, more research is needed to obtain deeper insight into this dynamic.

Both leaders and employees provided positive evaluations of reciprocal trust, in particular those who worked mainly in remote contexts or led remote employees. However, nearly a third of the participants also reported that the advent of remote leadership has weakened the workplace atmosphere, and a fifth of them reported decreased opportunities to make contact with their leader. The latter aspect is in line with the results of our qualitative data as well as with the results presented by Ameel *et al.* (2022), who found that remote leadership is perceived to distance leaders from the clinical setting. Therefore, the presented findings provide insight into how leaders and organizations must proactively consider how remote leadership is enacted to mitigate possible negative consequences, such as decreased trust and adverse working outcomes (Terkamo-Moisio *et al.*, 2022). Potential solutions to the decreased accessibility of leaders could be open calendars, publishing regular contact hours in which the leader has no meetings, and providing various ways in which employees can contact their leader. Furthermore, leaders should strive to show empathy and interest in their employees, both of which have been shown to strengthen team culture and reciprocal trust in the remote context (Terkamo-Moisio *et al.*, 2022). Leaders should also ask for organizational support to strengthen and develop their communication skills. Furthermore, leaders should consider the importance of regular informal communication with their employees (Kiljunen *et al.*, 2022; Terkamo-Moisio *et al.*, 2022), as over a fifth of the participants in this study reported that remote leadership and digitalization have weakened leader–employee communication.

### Strengths and limitations

Collecting a sample from one entire health and social service organization and a good response rate (56%) among the leaders strengthened this study. However, the response rate among employees was low (13%), which is a limitation and decreases the generalizability of the presented results. One possible reason for the low response rate among employees may be the timepoint of data collection, as the hugely increased workload and burden due to the COVID-19 pandemic may have hindered or completely prevented employees’ participation. Furthermore, health and social care is known to be a human-intensive field, one in which remote leadership emerged and received increased attention during the COVID-19 pandemic (Kiljunen *et al.*, 2022; Terkamo-Moisio *et al.*, 2022). It is possible that the employees felt too inexperienced or did not regard the topic as relevant for themselves, and this may have contributed to a low response rate. The total sampling enhances the generalizability from the perspective of leadership development. Less than half of the participants worked mainly in remote contexts as remote leaders or remote employees, which may be considered to affect the reliability of the results. However, comparison of the groups did not reveal statistically significant differences in the opinions expressed, with the exception of the three statements that are addressed in the discussion. Furthermore, participants may have had some experience, and they may have formed an opinion about remote leadership even though they mainly work in a traditional, face-to-face context. The used questionnaire was designed with the assistance of a group of experts; furthermore, it was pretested by 61 participants – both aspects strengthen the reliability of this research. The data were collected with a self-reported questionnaire, which may be seen as a further limitation due to the possibility of response bias driven by overestimation of ability and/or providing socially desirable responses (Rosenman *et al.*, 2011). Nevertheless, it can be assumed that the anonymity of participation would decrease the probability of response bias. The qualitative data provided rich insights into the phenomenon of remote leadership. It is noticeable that participants wrote their descriptions in a more condensed manner compared with, e.g. semi-structured interviews, which may be seen as a general limitation of open-ended questions in a survey (Burns and Grove, 2009). The qualitative analysis was independently carried out by two members of the research team, which strengthens the credibility of the presented findings. Credibility was further strengthened by the provision of detailed descriptions of the participants and the research context, while authenticity was strengthened by including participants’ original expressions in the text (Elo *et al.*, 2014).

### Implications for practice and future studies

This research, which was carried out in a single large health and social care organization, revealed that remote leadership is conducted in various ways and results in different kinds of experiences among leaders and employees. Only some of them recognized that their organization to have guidelines for remote leadership. Therefore, organizations should critically assess their practices of remote leadership and strive to develop guidelines by involving different stakeholders to support their leaders and employees who work in remote contexts, as such guidelines have been acknowledged to be central for successful remote leadership. Furthermore, organizations should ensure that information confirming the existence of these guidelines is distributed among the employees and that it is easily accessible in order to increase the employees’ awareness. In addition, the development of an instrument for measuring remote leadership would enable the observation and evaluation of this developing form of leadership.

The challenges related to trust and interaction in a remote context are present in the results of the current results. To tackle these issues, remote leaders should be offered regular possibilities to strengthen their skills related to communication and digital communication tools. Participants’ perceptions of task-oriented work and one-way information flows, as well as the lack of employees acknowledging the prevalence of this approach, should compel organizations to consider and clearly address the kind of leadership philosophy to which they are committed. Furthermore, scientific knowledge is needed to reveal more about how remote leadership is understood and conceptualized.

Current research interest in remote leadership is focusing on issues such as communication and trust in the remote context, with particular emphasis on the leaders’ perspectives. Less is known about the views of employees on remote leadership and its associations with, for example, work-related wellbeing or the burden imposed by digitalization; these aspects should be considered in future research. In addition, the comparisons of the benefits of traditional and remote leadership could provide important insights for the evidence-based development of hybrid leadership, which has been identified as a future trend for the industry.

## Conclusions

The remote context increases the significance of leadership but also carries the risk of development in the direction of task-orientation rather than people-centered leadership within the health and social care sector. Preventing this requires attention from organizations and future research based on solid evidence. Education is one means to prevent the negative effects of remote leadership, such as digitalization-related burdens or work-related uncertainty. Therefore, leaders and employees working mainly – or even just occasionally – in remote contexts should be offered regular opportunities to enhance their digital and communication skills; this could ensure successful collaboration and interaction. Organizations should also create and strengthen psychologically safe environments and structures of participation to enhance their employees’ sense of community.

## Figures and Tables

**Table 1.** Participants’ demographic information

Demographic information	*n*	%
*Gender* (*n* = 221)		
Male	13	6
Female	206	93
n/a	2	1
*Age in years* (*n* = 215)		
≤ 40	39	18
41–55	76	35
≥ 56	100	47
*Educational level* (*n* = 221)		
Master’s degree or higher	43	19
Bachelor’s degree or equivalent	114	52
Secondary education or other	64	29
*Works mainly in remote context* (*n* = 215)		
As remote leader	16	7
As remote employee	69	32
Does not apply to me	130	61
*Professional status* (*n* = 222)		
In management position	45	20
Employee	177	80
*Number of employees* (*n* = 35)		
≤15	19	54
≥16	16	46
*Leader of remote employees* (*n* = 28)		
No	13	46
Yes	15	54

**Source:** Derived from analysis of the data

**Table 2.** Participants’ perceptions of remote leadership

Subcategories	Upper categories	Main category
Successful and functioning leadership	Appearance of remote leadership	Participants’ perceptions of remote leadership and its development
Distanced and authoritarian leadership		
Inappropriate for the own work unit		
Reduced into virtual meetings		
Developing form of leadership		
Part of daily work life	Experiences of remote leadership	
One-way flow of information		
Electrical information which is challenging to access		
Active and leader-driven remote leadership	Development of remote leadership	
Significance of face-to-face meetings		
Regular interaction and information		
Education of leaders and employees		

**Source:** Derived from the inductive content analysis of the data

**Table 3.** Health and social care leaders’ and employees’ perceptions of remote leadership

Statement	Agree		Neutral		Disagree	
	*n*	%	*n*	%	*n*	%
The importance of management has increased with the increased prevalence of remote leadership (*n* = 209)	142	68	42	20	25	12
I feel that remote leadership has not weakened my opportunities to be in contact with my manager (*n* = 198)	107	54	51	26	40	20
The spread of digitization burdens me as an employee (*n* = 212)	93	44	36	17	83	39
Physical distance has weakened the trust between my manager and I (*n* = 201)	46	23	64	32	91	45
The spread of digital communication tools has weakened interactions between my manager and I (*n* = 207)	48	23	53	26	106	51
Remote leadership has had a positive effect on the working atmosphere (*n* = 199)	42	21	97	49	60	30
I do feel uncertain about the continuation of my employment as remote leadership increases (*n* = 182)	21	12	41	22	120	66

**Source:** Derived from analysis of the data

**Table 4.** Health and social care leaders’ perceptions of remote leadership

Statement	Agree	Neutral	Disagree
	%		
I treat all my employees equally regardless of physical distance (*n* = 39)	94	3	3
There is mutual trust between my employees and me (*n* = 39)	87	13	–
My leadership skills meet the requirements of remote leadership (*n* = 38)	74	13	13
I feel that I am sufficiently available to my employees despite the physical distance (*n* = 39)	74	13	13
Increased digitalization has made work as a leader easier (*n* = 39)	64	21	15
Our organization has common rules of the game for remote leadership (*n* = 39)	59	20	21
Increased digitalization burdens me as a leader (*n* = 39)	31	13	56
Remote leadership has increased insecurity among employees (*n* = 38)	26	32	42
Remote leadership communication tools weaken interactions (*n* = 39)	18	33	49
Physical distance has weakened the trust between my employees and I (*n* = 39)	15	23	62
Employees have unrealistic expectations of remote leadership (*n* = 39)	13	36	51
Remote leadership makes me feel insecure as a leader (*n* = 39)	3	20	77

**Source:** Derived from analysis of the data
